# A Sequential Multiple Assignment Randomized Trial of scalable interventions for ART delivery in South Africa: the SMART ART study

**DOI:** 10.1186/s13063-022-07025-x

**Published:** 2023-01-17

**Authors:** Alastair van Heerden, Adam Szpiro, Xolani Ntinga, Connie Celum, Heidi van Rooyen, Zaynab Essack, Ruanne Barnabas

**Affiliations:** 1 Centre for Community Based Research, Human Sciences Research Council, Pietermaritzburg, South Africa; 2 MRC/Wits Developmental Pathways for Health Research Unit, University of the Witwatersrand, Johannesburg, South Africa; 3 Department of Global Health, University of Washington, Seattle, WA USA; 4 Division of Allergy and Infectious Diseases, University of Washington, Seattle, WA USA; 5 Human Sciences Research Council, Pietermaritzburg, South Africa; 6 Division of Infectious Diseases, Massachusetts General Hospital, Boston, MA USA; 7 Harvard Medical School, Boston, MA USA

**Keywords:** HIV treatment, South Africa, Client-centered, Differentiated service delivery (DSD), Sequential multiple assignment randomized trial (SMART)

## Abstract

**Background:**

Of the 8 million people in South Africa living with HIV, 74% of persons living with HIV are on antiretroviral therapy (ART) and 65% are virally suppressed. Detectable viral load results in HIV-associated morbidity and mortality and HIV transmission. Patient barriers to care, such as missed wages, transport costs, and long wait times for clinic visits and ART refills, are associated with detectable viral load. HIV differentiated service delivery (DSD) has simplified ART delivery for clients who achieve viral suppression and engage in care. However, DSD needs adaptation to serve clients who are not engaged in care.

**Methods:**

A Sequential Multiple Assignment Randomized Trial will be undertaken in KwaZulu-Natal, South Africa, to test adaptive ART delivery for persons with detectable viral load and/or who are not engaged in care. The types of differentiated service delivery (DSD) which will be examined in this study are clinic-based incentives, community-based smart lockers, and home delivery. The study plans to enroll up to 900 participants-people living with HIV, eligible for ART, and who are not engaged in care. The study aims to assess the proportion of ART-eligible persons living with HIV who achieve viral suppression at 18 months. The study will also evaluate the preferences of clients and providers for differentiated service delivery and evaluate the cost-effectiveness of adaptive HIV treatment for those who are not engaged in care.

**Discussion:**

To increase population-level viral suppression, persons with detectable viral load need responsive DSD interventions. A Sequential Multiple Assignment Randomized Trial (SMART) design facilitates the evaluation of a stepped, adaptive approach to achieving viral suppression with “right-sized” interventions for patients most in need of effective and efficient HIV care delivery strategies.

**Trial registration:**

ClinicalTrials.gov NCT05090150. Registered on October 22, 2021

## Administrative information

Note: the numbers in curly brackets in this protocol refer to SPIRIT checklist item numbers. The order of the items has been modified to group similar items (see http://www.equator-network.org/reporting-guidelines/spirit-2013-statement-defining-standard-protocol-items-for-clinical-trials/).

**Table:** 

Title {1}	A Sequential Multiple Assignment Randomized Trial of scalable interventions for ART delivery in South Africa: the SMART ART Study
Trial registration {2a and 2b}.	https://clinicaltrials.gov/ct2/show/NCT05090150
Protocol version {3}	Version 1.2, July 9th, 2021
Funding {4}	US National Institutes of Health (R01MH124465T)
Author details {5a}	Alastair van Heerden^5,7^, Adam Szpiro^,3,4^, Xolani Ntinga^5^, Zaynab Essack^5^, Connie Celum^,3,4^, Heidi van Rooyen^,6,7^, Ruanne Barnabas^1,2^ ^1^*Division of Infectious Diseases, Massachusetts General Hospital, Boston, MA, US* ^2^*Harvard Medical School, Boston, MA, US* ^3^*Department of Global Health, University of Washington, Seattle, WA, US* ^4^*Department of Medicine, Division of Allergy and Infectious Diseases, University of Washington, Seattle, WA, US* ^5^*Centre for Community Based Research, Human Sciences Research Council, Pietermaritzburg, South Africa.* ^6^*Human Sciences Research Council, Pietermaritzburg, South Africa.* ^7^*MRC/Wits Developmental Pathways for Health Research Unit, University of the Witwatersrand, South Africa*
Name and contact information for the trial sponsor {5b}	Massachusetts General Hospital Dr. Ruanne Barnabas 55 Fruit Street Boston, MA 02114, United States
Role of sponsor {5c}	The funder of the study had no role in study design, data collection, data analysis, data interpretation, or writing of the manuscript.

## Introduction

### Background and rationale {6a}

HIV care programs have evolved into a chronic disease care model in South Africa, where eight million people are living with HIV [1]. Antiretroviral therapy (ART) has dramatically impacted individual life expectancy; since the rollout of ART, life expectancy has increased by 15.2 years among men and 17.2 years among women [2]. Furthermore, ART reduces the risk of HIV transmission, which has slowed the epidemic growth and decreased HIV incidence. In South Africa, 74% (5.5 million) of persons living with HIV are on ART and 65% (4.9 million) are virally suppressed [3]. Detectable viral load carries the risk of morbidity and mortality from opportunistic infections and HIV-associated diseases. Mortality associated with mid-adulthood carries a high social and economic cost to society. In addition to the high individual benefit of treating HIV, expanding ART coverage is also a high-value public health intervention due to gains in life years. For individual health- and population-level benefits, strategies are needed to sustain the high level of viral suppression for the lifetime of persons living with HIV. Differentiated service delivery (DSD) is an approach that addresses barriers and closes gaps.

Differentiated service delivery (DSD), also known as differentiated care, for HIV is a client-centered approach that simplifies and adapts HIV services across the HIV care continuum, in ways that both serve the needs of people living with HIV better and reduce unnecessary burdens on the health system [4]. DSD models may alter the provider, intensity, location, or frequency of ART services for specific populations [5] and seek to allocate resources more effectively by tailoring delivery strategies to the needs of diverse groups of clients.

DSD models have been implemented across sub-Saharan Africa that differ from standard clinic-based care and are targeted to stable patients (i.e., clients with suppressed viral loads) on ART [6, 7]. Models are classified into facility-based models that leverage existing infrastructure and community-based models that deliver ART closer to clients [8]. Examples of DSD models include multi-month prescribing, task shifting, incentives, community ART, and home delivery.

These DSD models differ in the intensity of service provision and, consequently, in the resources required. For example, lottery incentives are low cost to implement but have a short-term effect increasing ART initiation but not long-term viral suppression [8]. Community ART provides decentralized ART delivery outside the clinic, and home ART delivery takes ART to clients’ homes. Community and home ART delivery work as well as clinic-based services to achieve viral suppression for stable clients [9, 10] are likely to be more costly than clinic-based services; however, for clients with a detectable viral load, they can overcome individual and structural barriers to clinic visits and the health benefits could outweigh increases in cost but requires evaluation.

The simplification and client-centeredness of DSD approaches could be adapted to help those who are struggling instead of succeeding. As HIV services, specifically supplying ART and conducting monitoring, mature, the most pressing need is for clients to access ART over their lifetimes—i.e., services to support lifelong retention. However, 5-year retention in care in South Africa is 60% [11–14]. As currently implemented, DSD is not working to increase overall viral suppression; but since it is offered to stable clients only, there is little room for improvement. Recently, the impact of adherence clubs and decentralized medication delivery was assessed in a cluster randomized trial in South Africa which found no difference in viral suppression between standard clinic care and adherence clubs or decentralized medication delivery [15]. Also, community and home ART delivery showed no difference in viral suppression compared to clinic ART [9, 10]. Furthermore, for unstable patients, enhanced adherence counseling and tracing of clients who missed visits showed no difference in rates of viral resuppression [15]. The innovations of DSD could be extended to clients not engaged in care.

**Fig. 1:**
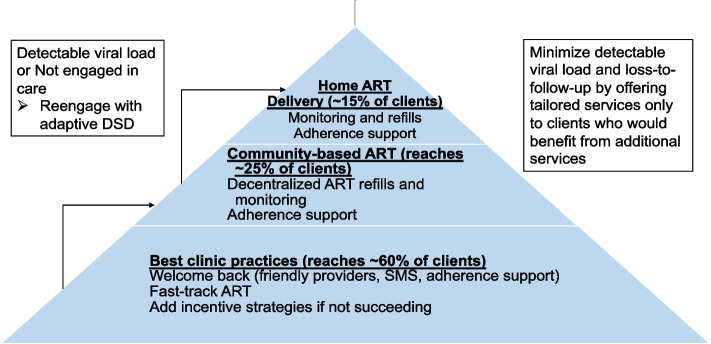
“Right-sizing” DSD to fit client needs and provider services so that more intensive services reach clients that would benefit from simplification and client-centered approaches

We and others found that DSD, as currently implemented, often costs effectively the same as standard models [16]. The annual cost per patient within DSD models (excluding drugs) ranged from $27 to $889 (2018 USD). Of the 11 DSD models reporting incremental costs, seven found DSD to be cost-saving. Personnel was the most common driver of reduced costs, but higher utilization sometimes offset savings. Adaptive DSD could provide a stepped approach for clients who are not succeeding in standard care models to access care outside the clinic (Fig. 1). This would be in contrast to requiring persons not succeeding at the clinic to remain at the clinic and possibly have even more appointments for enhanced counseling. Existing services work for more than half of South African clients. But the remainder of clients require an approach that uses routinely collected laboratory testing (viral load), pharmacy/clinic data on missed refills and missed appointments, and client-reported barriers to offer services outside the clinic rather than limiting DSD to stable patients. Clients who report barriers to engaging in care have a threefold higher risk for HIV-associated mortality [17]. Adapting DSD to meet the needs of these clients who are not succeeding in standard clinic models has the potential to efficiently increase viral suppression and be cost-saving (due to averted costs of morbidity, mortality, and transmission) but requires evaluation.

A unique opportunity exists to evaluate the impact of strategies to reengage and retain people living with HIV with detectable viral load and/or otherwise not engaged in care, specifically by providing enhanced services to clients with detectable viral load and/or who are not engaged in care. To address gaps in care, the South African National Department of Health (NDOH) clinics are rolling out interventions to increase viral suppression and retention in care. For example, Médecins Sans Frontières (MSF) has piloted a “Welcome Back” service at HIV clinics in the Western Cape to reengage clients in care who have previously been lost to follow-up or who have detectable viral load and this service is being further refined and adapted at other clinics. The Welcome Back service has three pillars: (1) identification and linkage, (2) medical support including care for advanced HIV, and (3) psychological support including adherence support. Working with MSF, we will adapt their clinic staff training and client materials to support best clinic practices. In the proposed study, we will work with clinic teams to implement the MSF Welcome Back services and other evidence-based clinic interventions, specifically adequate stock of ART, fast-track ART, time for clients, and kind providers which have been associated with higher retention in care [18, 19] to maximize the impact of clinic-based services. The rationale for strengthening clinic services is to provide the best possible opportunity for standard services to work for clients.

DSD approaches work for persons not engaged in care. Building on best clinic practices, lottery incentives shorten time to ART initiation [8] and are low cost to implement. Community and home ART remove logistic barriers to clinic access and have been shown to increase viral suppression rates among men [20]. A systematic review found that community healthcare worker-led inventions, such as community ART, significantly improved viral suppression by 40% among participants not suppressed [21]. By providing evidence-based DSD interventions for clients not virally suppressed despite best clinic practices, we will test the impact of adaptive DSD among individuals who may benefit the most. We propose to use a Sequential Multiple Assignment Randomized Trial (SMART) design [22], individuals who fail best clinic practices with and without lottery incentives will be rerandomized to either community ART (e.g., retrieving one’s ART from a smart locker), home ART delivery, or continuation in the standard clinic group. Stepped care involves a sequence of decisions based on the individual’s outcome, altering the intensity and type of intervention over time if the person is not responding. A SMART design provides high-quality data that can be used to construct adaptive interventions and is well suited for this study. Specifically, the adaptive intervention can be operationalized to maximize the long-term primary outcome, i.e., viral suppression.

## Objectives {7}

Our clinical trial aims to determine whether community-based ART initiation and maintenance increase the proportion of ART-eligible persons living with HIV who achieve viral suppression at 18 months. We will also evaluate as secondary objectives the (1) qualitative preferences of clients and providers for differentiated service delivery intensification and (2) cost, budget impact, and cost-effectiveness of an adaptative HIV treatment strategy for persons living with HIV who are not engaged in care to support viral suppression.

## Trial design {8}

**Fig. 2:**
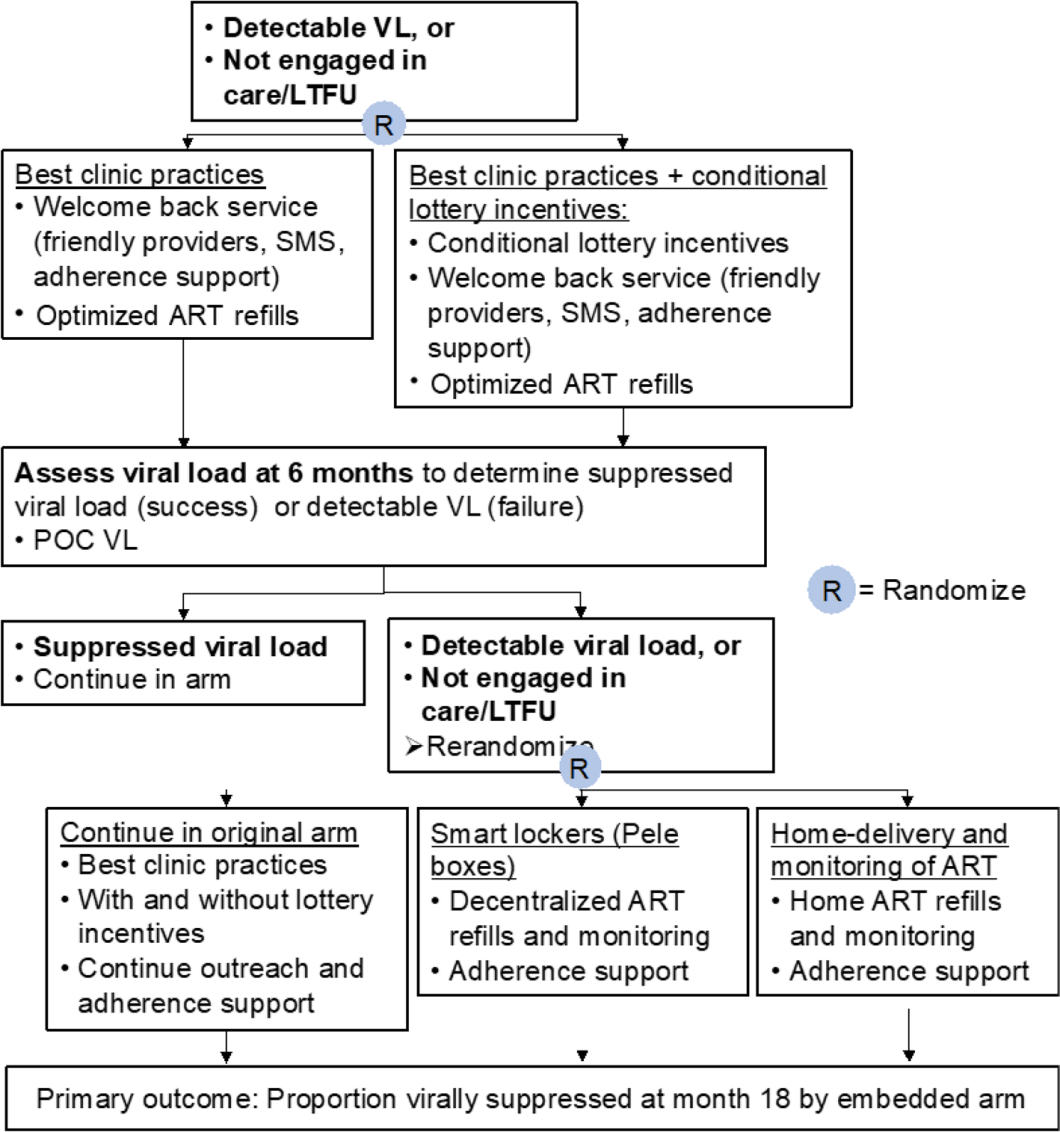
SMART ART trial design

The SMART ART study is a Sequential Multiple Assignment Randomized Trial (SMART) to test an adaptive treatment strategy for persons living with HIV who are not virally suppressed within existing clinic services (Fig. 2). Persons with detectable viral load or not engaged in care for 6 months will be randomized 1:1 to standard of care with or without lottery incentives for linking to care. Participants who continue to have a detectable viral load or not engaged in care 6 months after the first randomization will be further randomized (2:1:1) to either continue in their original randomization group, smart locker ART pickup, or home ART delivery. Follow-up will continue for 12 months following the rerandomization, with a total follow-up time of 18 months.

## Methods: participants, interventions, and outcomes

### Study setting {9}

Greater Edendale Area within the Umgungundlovu District in KwaZulu-Natal, South Africa.

### Eligibility criteria {10}

To be eligible, participants

•Age 18 years or older

•Able and willing to provide informed consent for study procedures

•Intend to reside in the study community for the duration of follow-up

•Living with HIV and eligible for ART by national guidelines

- Have a detectable viral load greater than the lower limit of detection and/or not engaged in care
- Are stable clinically (CD4>100 cells/μL, no moderate/severe screening laboratory abnormalities for kidney function, not receiving treatment for active tuberculosis or other opportunistic infections)

There are no separate exclusion criteria.

### Who will take informed consent? {26a}

A study counselor will review the study information sheets (screen consent and when applicable enrolment consent) with all potential participants and discuss emerging questions with the individual. In the event that informed consent is being discussed with a couple rather than an individual, each partner will be asked to provide separate independent informed consent for study participation. If the participant is illiterate, a witness will be present when informed consent is verbally administered and a checkmark is obtained. If electronic consent is unavailable due to technical challenges, then paper back consents will be used. Separate electronic informed consents complying to ICH guidelines E6 4.8. will be completed for screening and for enrolment of eligible participants.

### Additional consent provisions for collection and use of participant data and biological specimens {26b}

All participants completing the enrollment consent are given the option of agreeing to future contact for either new research protocols or to receive general information about research findings. Participants are also asked whether they agree or not to be contacted by text message or phone for appointment reminders and receiving of test results.

## Interventions

### Explanation for the choice of comparators {6b}

The innovation advanced in this study is testing adaptive DSD strategies. The “supply (dissemination) – demand (diffusion) – infrastructure (delivery) model” provides a useful framework to organize activities and bridge the translational research-practice gap [23]. “Supply” activities focus on users, both care provider and clients, by training, documenting, improving, and communicating about adaptive DSD. “Demand” involves both users and clinical implementers by building demand through an analysis of potential adopters’ decision-making and perceptions of an innovation’s pros and cons. “Infrastructure” focuses on delivery obstacles, including building capacity of systems to deliver the intervention. Key questions for adaptive DSD are, therefore, (1) evaluate the impact of offering more intensive, adaptive services to persons not succeeding in standard clinic care; (2) working closely with communities, clients, and providers, identify and address concerns for implementation; and (3) estimating the budget impact and cost-effectiveness. We used this approach to select our comparator strategies for this adaptive DSD trial which included standard facility care and lottery incentives for clinic linkage in the first randomization and home delivery and smart delivery lockers placed in the community at the second randomization time point.

### Intervention description {11a}

We will conduct a Sequential Multiple Assignment Randomized Trial (SMART) with participants presenting with a viral load above the limit of detection for the assay or who are not engaged in care/lost to follow-up (LFTU). To classify as not engaged, participants must meet one of the following criteria: (1) more than 6 months have passed since they were referred to the clinic and they have not attended, (2) they have missed a clinic appointment and have not rescheduled, (3) they have missed ART pick-ups without re-attempting pick-up, (4) they are on the clinic list for tracking, or (5) they report two or more barriers to engaging in standard clinic-based care (logistics including clinic hours, transportation costs, and wait times, stigma, not wanting to visit the clinic, and concerned about being treated with respect at the clinic).

#### Participants randomized to best clinic practices without lottery incentives

Enrolled participants will first be randomly assigned to low-intensity interventions: either best clinic practices or best clinic practices with a conditional lottery incentive. Participants randomized to the standard of care (SOC) best clinic practice group receive a clinic referral letter including the date of the HIV test, information on the clinical screening, and that they are being referred to the clinic for ART initiation. The study team will work with the clinics in the study area to provide training on the “Welcome Back” Campaign that was launched by the South African Department of Health in December 2020 on World AIDS Day. The Welcome Back service is a model of HIV care that aims to improve engagements in HIV services and promote long-term retention and care. The program focuses on those patients that leave care and return to care on their own and ensures that they are welcomed back to the clinic in a non-judgmental way. Welcome Back services include text message reminders/outreach; optimized ART pharmacy refills, including fast-track refills and multi-month dispensing as provided by the clinic; and adherence support. Participants are asked to notify the study team when they start ART, and receive phone calls every 3 months to assess linkage to care. Participants will schedule their month 6 follow-up visit to determine if rerandomization is required.

#### Participants randomized to best clinic practices with lottery incentives

Participants randomized to the SOC with lottery incentives group will receive a clinic referral letter including the date of the HIV test, information on the clinical screening, and that they are being referred to the clinic for ART initiation. Participants will be asked to scan their barcode at the clinic or notify the study team of their visit and the reason for the visit (ART initiation, refill, or follow-up). Participants will be notified by text message that they have been entered into the lottery, and within a week, they will be notified whether they have won. All participants will be notified of when there is an (anonymous) lottery winner. Lottery winners are predetermined through a random selection to ensure that winning may occur in a timely manner linked to behavior. If participants do not notify the study or scan their barcode, at their quarterly phone call follow-up, they would be entered into the lottery if they are eligible. The lottery prize will be a pre-loaded smart phone or equivalent with an approximate value of 1000 ZAR (~71 USD). Participants also will receive phone calls after 3 months to assess linkage to care. Participants will schedule their month 6 follow-up visit to determine if rerandomization is required.

#### Six-month study visit to determine eligibility for the second randomization

After the initial 6-month stage, a visit will take place at a location of the participant’s choice with options for the visit to take place at home, at a mobile van, a location in the community, or at the clinic. Participants will complete questionnaires to assess engagement in care and barriers to accessing care. To further determine eligibility, specimens will be collected for viral load testing (either plasma or point-of-care viral load testing, if available). Participant clinic and pharmacy records will be checked for missed clinic appointments and/or missed ART refills. Participants who are virally suppressed and engaged in care at month 6 will continue in their original group, receive quarterly phone calls, and schedule their exit visit at month 18. Locator information will be confirmed. Participants who meet the criteria for not engaged in care or LTFU will receive their second randomization once the questionnaire is complete. Viral load results will be provided either immediately if using a point-of-care test or within a week to facilitate rerandomization for participants who are engaged in care. Once it has been determined that the participant is eligible to be rerandomized, the first step will be to determine whether or not ART has been initiated. If the participant is not engaged in care but ART has been initiated, the clinical screen will be conducted to exclude symptoms of active TB or cryptococcal meningitis, point-of-care creatinine will be checked, and a 3-month refill will be provided. Participants will be taught to self-collect DBS for viral load testing at this visit.

#### Participants initiating ART at the month 6 visit

For participants requiring ART initiation, a study nurse will initiate ART at home or in a mobile van. The study nurse will review the participant’s eligibility for ART initiation at home, provide counseling on adherence and adverse effects, and will dispense the first 90 days of medication. The study staff member will register the participant at the clinic so that the participant records are available at the clinic should the participant seek care there for any reason. The registration will either be done electronically or through paper forms, depending on the clinic capacity to receive electronic referrals. If appropriate, the participant may be transferred to the HSRC via Tier.net. Participants will receive a phone call 7 days and 30 days after initiating ART to complete a standard symptom screen for ART adverse effects and referred to clinic care if necessary, following the procedures detailed below under “Referral to the clinic for symptoms and clinical events.” If indicated, tuberculosis (TB) preventive treatment (TPT) and trimethoprim-sulfamethoxazole will be provided to prevent TB and pneumocystis pneumonia.

#### Community (smart locker) delivery

If participants are randomized to the community (smart locker) delivery group, they will receive ART refill delivery via a smart locker in a secure location. They will complete their clinical screen via telehealth, including an option for a secure video link, and self-collect specimens for viral load screening. If the participant is not able to access the smart locker due to the geographic location, mobile smart lockers will be provided via a mobile van at the time of delivery.

#### Home ART delivery

Participants randomized to the home ART delivery group will receive ART delivery at home or at a location of their choice, such as a community center. At ART delivery visits, a clinical screening questionnaire will be completed, and blood collected for viral load and creatinine tested as indicated by the clinical monitoring schedule following local guidelines. These procedures will be conducted by a study team member in person, with oversight from a nurse, or via telehealth with a nurse. If no-contact delivery is necessary due to COVID-19 restrictions, the participant will self-collect DBS for viral load testing and provide that when the medication is delivered. Participants will have a home delivery appointment scheduled 3 months after ART initiation.

### Criteria for discontinuing or modifying allocated interventions {11b}

Participants will be encouraged to visit the clinic for medical concerns outside of ART monitoring and adherence counseling. During participant ART resupply and monitoring visits, they will complete a standardized symptom screening questionnaire for adverse effects of ART, tuberculosis, opportunistic infections, and STIs. Furthermore, all participants on TDF/FTC or 3TC/TDF will receive the standard ART lab monitoring, including a creatinine test to monitor their renal function. Participants who have severe (grade 3/4) adverse effects, according to the DAIDS guidelines and serious adverse effects, will be referred to the clinic for medical evaluation and appropriate diagnostic testing. We have established relationships with the clinics who expect to see study participants. All participants who experience adverse events will receive follow-up until the adverse event is resolved. The SMART ART study clinical monitor, based at the University of Washington Coordinating Center, will review all severe (grade 3/4) and serious adverse events to ensure follow-up and reporting. Participants who have two viral loads with >1500 copies/mL will be referred to the participating local HIV clinic(s) for evaluation for drug resistance and initiation of second-line ART. Participants who are referred back to the clinic will continue to receive their study follow-up visits and can return to home delivery or smart locker delivery with ART monitoring and refills once they are clinically stable.

### Strategies to improve adherence to interventions {11c}

Participants will receive a non-identifying text message within 1 week of viral load testing to communicate that “all is going well” (i.e., if their viral load is suppressed), contacting them to have additional adherence counseling—“contact us for more information” (i.e., if first viral load not suppressed), or asking them to come into the clinic for assessment for treatment failure (i.e., if two viral loads are not suppressed following intervening adherence counseling). Adherence counseling for participants with VL>1500 copies/mL will identify barriers and strategies to increase ART adherence.

### Relevant concomitant care permitted or prohibited during the trial {11d}

ART, TPT, and other chronic meds will be dispensed.

### Provisions for post-trial care {30}

Participants who suffer harm from participating in this study will be offered a referral to one of the study site’s referral partners or, if appropriate, care at the HSRC site clinic, free of charge. No monetary compensation will be provided. If a participant requires medical care that the study clinic cannot provide, the study doctors will refer participants to the appropriate services or organizations that can provide care for the injury or harm.

### Outcomes {12}

The primary outcome of this trial is viral suppression at 18 months, comparing (i) all individuals randomized to clinic-based care with or without lottery incentives for the full 18 months and (ii) nonresponding individuals rerandomized to home- or community-based care to those rerandomized to clinic-based care. The secondary outcomes are (a) retention in care (defined as the proportion of clinical visits and medication refills missed over the last 12 months of the intervention) and (b) time to ART initiation, and additional analyses will include (c) impact of the adaptive interventions among men; (d) impact among the two subgroups at enrollment (persons with detectable viral load and persons not engaged in care); (e) assessment of how baseline variables influence the difference between adaptive interventions; and (f) comparison of viral suppression and retention outcomes between local clinics. For home delivery, missed ART deliveries, total miles traveled, and CO^2^ production will also be measured to quantify costs.

### Participant timeline {13}

**Fig. 3:**
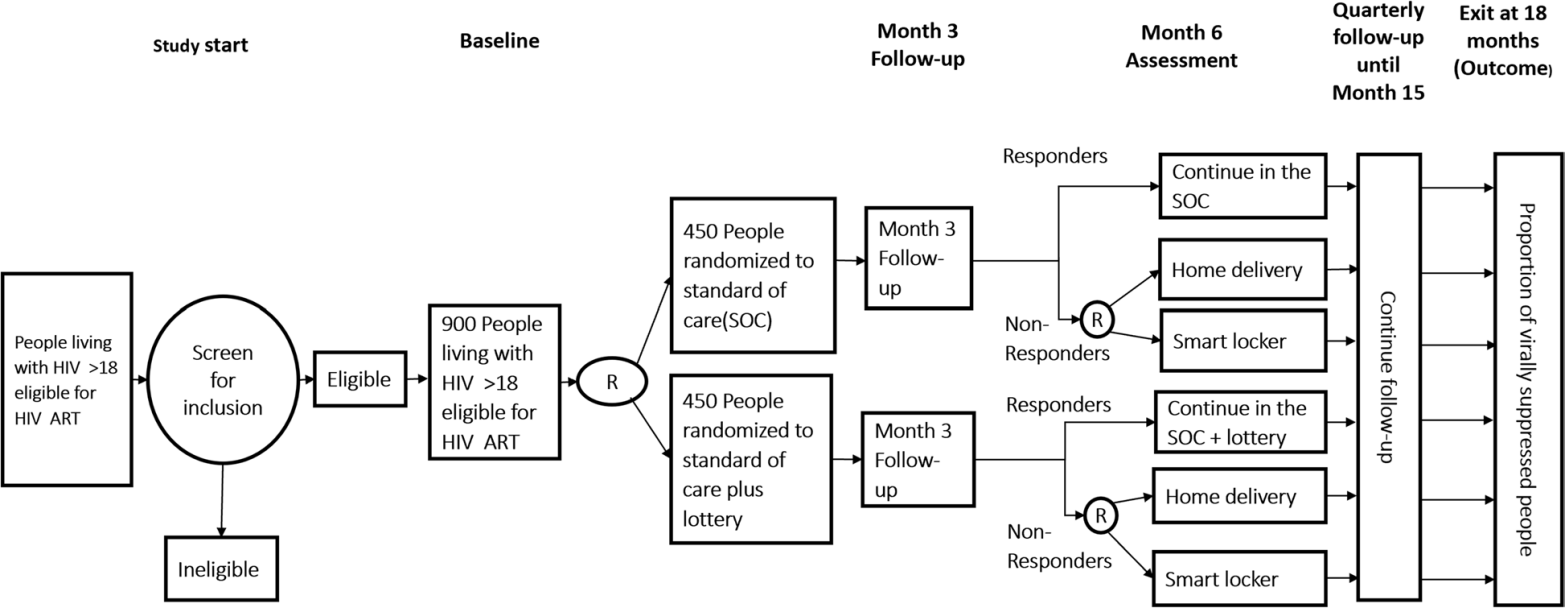
The flow of participants through the study at associated time points

The flow of participants through the study at associated time points is presented in Fig. 3.

### Sample size {14}

With a sample size of *N*=900 persons, randomized 1:1, there will be *n*=450 each in the best clinic practices and best clinic practices with incentives groups. We expect 30% to be virally suppressed in the clinic group and 45% in the best clinic practices plus incentives group at 6 months. At the next randomization step, assuming 95% retention, we expect approximately 520 participants to be randomized (2:1:1) to continue in the original group, community ART (smart lockers), or home ART delivery. For the first comparison, clinic-based ART compared to clinic-based ART plus incentives, we have 79% power to see a 15% difference in viral suppression between the groups at 6 months. For the comparison of either home or community ART vs. best clinic practices, we would have 99% power to see an approximately 50% difference in viral suppression between the groups. For the secondary comparison, home ART versus community-based ART, we have 90% power to see a 20% difference in viral suppression—50% in the community ART group and 70% in the home ART delivery group at 18 months. In previous studies in the same site communities, we have achieved retention rates between 98 and 100% at 12 months, demonstrating that we can minimize loss to follow-up in the SMART ART clinical trial. Thus, overall, we will be well powered to see differences of clinical significance—an absolute 40% increase in viral suppression will achieve the UNAIDS goal for 2025 and the initial investment offset by healthcare costs averted of advanced HIV and transmission.

### Recruitment {15}

Prior to recruitment, community mobilizers will visit communities to provide details about the study including the community-based HIV testing and approximate dates available in that community and at the clinic. Site teams are experienced in HTC and are well known to the clinic staff and community leaders. We will identify participants for recruitment through two means: (1) clinic recruitment and (2) community-based HIV testing and counseling through home and mobile testing.

#### Recruitment at HIV clinics

Outreach for recruitment will be conducted by study staff at HIV clinics to identify persons living with HIV who are presenting at the clinic for the first time and are not yet engaged in care. Participants recruited at the clinic will be eligible to continue with study procedures outlined below. For Clinic Outreach Team recruitment, we have worked closely with the Caluza Clinic who have identified clients who have had a detectable viral load and/or who have not been engaged in care. Under new guidance to offer “Welcome Back” services, clinic outreach teams will contact these clients to ensure that they are not engaged in care elsewhere. Clients will be informed about the study and referred to the SMART ART study team for information and, if they choose, study enrollment. Informed consent may take place at the clinic or at an alternate location of the participant’s choice, such as a community center.

#### Community-based HTC

##### Home HTC

Counseling and testing in home HTC is conducted in a private environment either inside the participant’s home or outside. During community mobilization, mobilizers will inform household members of the day and time of home HTC visits. For the home HTC visit, trained counselors will visit contiguous homes within the community.

##### Mobile HTC

Counseling and testing in mobile HTC is conducted in mobile testing centers in a private environment either inside the testing van or outside. Mobile HTC will be provided from mobile counseling and testing units and within suitable community venues identified during the community entry and consultation process. The community mapping process will be used to identify high-traffic spots in the community. This will include taxi ranks, popular shops, kiosks, weekly markets, communal service, and social venues such as churches, sports grounds, etc. A list of possible community testing venues will be developed and reviewed to ensure walking-distance coverage of all the settlement clusters in the community. These high-traffic spots will be used for mobilization activities. A suitable open space of land for the siting of the mobile testing unit will be identified proximal to the mobilization area. Where available and suitable, nearby community buildings or informal structures such as tents may be used to provide counseling and testing. Mobile HTC will be offered at high-traffic areas such as taxi ranks to identify persons who have not yet virally suppressed despite knowing their status and visiting the clinic in the last 6 months. Community-based HTC approaches for recruitment will allow us to recruit participants who do not come to the clinic despite knowing their status and clients who have attended the clinic but are not yet virally suppressed and engaged in care.

##### HIV self-testing (HIVST)

In order to further identify persons, particularly men, in the community who are less likely to participate in community-based HTC, we will provide HIV testing kits for self-administered testing and clear guidance for follow-up of persons electing to use the test kits. Persons who have a positive test using the kits will be invited to participate in SMART ART, receive confirmatory testing, and enroll in SMART ART if confirmed to be living with HIV. Kits for self-administered HIV testing (BioSure HIV Self Test, OraQuick, or other approved HIV Self Tests) will be distributed at the following venues: (1) HIV testing at the mobile van, which is often oversubscribed with some people having to wait or leave without a test, (2) providing HIV self-test kits to men and women in the SMART ART study to facilitate partner testing, and (3) HIV testing at outreach venues to reach men (men’s health events including multi-disease screening; peer ambassadors; work place testing; churches; music venues; and soccer games). Participants using self-test kits will watch a demonstration of the self-test kit, have the option of using a chatbot to help with self-testing, and receive information about the test kit, the need for confirmatory testing, and options for clinic-based services or SMART ART study enrollment to provide ART for persons testing positive. Persons electing to participate in self-testing will be encouraged to contact the study team for further information about linking to HIV treatment or prevention, including information about enrolling in the SMART ART study. Results of HIV self-testing will be confirmed with provider-conducted testing to determine eligibility in the SMART ART study.

## Assignment of interventions: allocation

### Sequence generation {16a}

We plan to enrol approximately 900 participants and randomize them initially at a ratio of 1:1 using varying size block randomization. At 6 months into the study, rerandomization of eligible participants will take place at a ratio of 2:1:1 again using varying size block randomization. Due to the intermittent availability of Internet access, envelopes will be used to randomize participants. Two recruitment teams will be used and therefore two different packets of envelopes were prepared, one set for each team.

### Concealment mechanism {16b}

The randomization sequence will be predetermined and available through sealed envelopes for each participant/household enrolled. The randomization group will not be determined until the participant has completed all screening procedures. The randomization will be designed at the University of Washington International Clinical Research Center (ICRC) Coordinating Center and will be provided via sealed envelopes, with UW ICRC oversight.

### Implementation {16c}

The allocation sequence will be generated by the unblinded biostatistician. The screening and enrolling nurse will assign participants who are eligible to the available interventions.

## Assignment of interventions: blinding

### Who will be blinded {17a}

Because of the difficulty masking study team and study participants to group allocation, the study is unblinded. However, staff assessing the primary outcome will be masked to the allocation of participants, as will study investigators.

### Procedure for unblinding if needed {17b}

This study is unblinded with respect to the study group.

## Data collection and management

### Plans for assessment and collection of outcomes {18a}

#### Screening and baseline

##### Screening questionnaire

After consent is obtained and locator information documented, the core set of risk assessment questions will be administered, including socio-demographic information; sexual and risk behavior; hypothetical risk and Belief in Medicines questionnaires; quality of life survey; knowledge, attitudes, and practices of HIV testing behavior; engagement in HIV treatment and prevention; clinical HIV history; costs of engaging in care; and related health activities. The staff member will administer the screening questionnaire to each individual in a confidential area.

##### Testing

Home testing and counseling (HTC) and mobile HTC will be conducted in communities in South Africa. Additional health services, specifically measurement of blood pressure and hemoglobin A1C testing for hypertension and diabetes screening, may be offered to all participants following local screening guidelines. Blood pressure will be measured two or three times, with a rest in-between reading, using an automated cuff and the lowest reading used to refer the participant for further medical care. Hemoglobin A1C will be the measured using a finger-prick specimen. Height and weight will be measured to calculate the body mass index of all participants. If BMI is measured, participants will receive information on maintaining a healthy weight through lifestyle interventions (e.g., diet and exercise). The screening results and referral to local clinics will be provided to participants who choose to engage in additional health screens.

##### HIV counseling and testing

Subsequently, the counselor will conduct individual counseling and HIV testing according to national guidelines. HIV results will be recorded. If necessary, for quality assurance (QA) of rapid HIV tests, dried blood spots will be collected. In the event that two individuals within a partnership test, the study staff will ask partners if they are interested in disclosing their test results to each other. Partners are encouraged to disclose their results, with the counselor facilitating disclosure, but are not required to disclose; for disclosure to take place, both partners must agree to disclose their results. If disclosure is agreed upon, the participants will be brought back together and disclosure counseling will occur. Once testing has been completed for a couple, and both members of the couple choose to disclose their results, disclosure counseling will be done. All HIV-negative participants will receive HIV prevention counseling and will be informed their participation in the study is complete. Participant who test negative for HIV may be referred to available HIV prevention studies. All participants will receive counseling on positive prevention and the benefits of engagement in HIV care. Persons living with HIV not on ART will continue with the screening process.

##### Screening for persons living with HIV not on ART

Persons living with HIV identified through home HTC, mobile HTC, or clinic recruitment and who are not on ART will be screened to stage their HIV infection by CD4 testing and the World Health Organization (WHO) symptomatic screen. To minimize the time to ART initiation, costs, and loss to follow-up, point-of-care tests will be used where possible, including for CD4 and creatinine.

##### CD4 testing

The CD4 test is a measure of immune function and is used to determine eligibility into SMART ART. Participants will be offered a point-of-care (POC) CD4 test, which will be obtained from a fingertip sample by lancet. If necessary for quality assurance (QA) of POC CD4 testing under field settings or if the POC CD4 test is not available, blood may also be collected at this time for standard laboratory CD4 count measurement by flow cytometry. The QA protocol is predetermined by the study sites to ensure reliable CD4 results.

##### WHO symptom screen and HIV engagement in care questionnaire

Participants will be screened for symptoms of tuberculosis, opportunistic infections (e.g., cryptococcal meningitis, PCP pneumonia), sexually transmitted infections, and clinical HIV through a set of standard WHO questions. Persons living with HIV will complete an HIV engagement in care questionnaire on previous HIV testing, CD4 count testing, clinical management, cost of engaging in care, ART use, and risk behavior.

##### Pregnancy testing

A urine pregnancy test will be performed, with urine collection done in a private setting. Women who have a positive pregnancy test will be eligible to participate in the study and will be linked to antenatal care at the clinic and followed until they link.

##### Creatinine testing

Persons living with HIV who are ART eligible will receive a point-of-care creatinine (Cr) test (using a lancet for finger-stick blood collection) to evaluate renal function for the common first-line regimens that include tenofovir (TDF) or tenofovir alafenamide (TAF). If an alternate first-line regimen is available following national treatment guidelines, appropriate screening will be conducted prior to ART initiation. A validated point-of-care test for monitoring creatinine will be used. If necessary for quality control (QC) of POC creatinine testing under field settings or if the POC creatinine test is not available, 5 mL of blood may also be collected at this time for standard laboratory creatinine measurement.

##### Tuberculosis (TB) testing

For persons reporting symptoms consistent of active TB, we will use a point-of-care TB test once available. The Cepheid GeneXpert test (Sunnyvale, CA) is approved for use in South Africa, and a portable version (Omni) is expected to be available. Both the GeneXpert and the Omni systems use sputum specimens (2 mL unprocessed sputum) that are processed for TB detection by Cartridge Based Nucleic Acid Amplification testing (CB-NAAT) using Xpert MTB/RiF assay technology. Persons who screen positive for active TB will be provided their test results and referred to the clinic or appropriate clinical study for further workup and treatment. Until this test is available, we will use symptom screening for active TB though it is non-specific and may exclude persons who are eligible for ART.

##### Cryptococcal testing

While ART is recommended for all persons living with HIV, for participants who have co-infections, ART initiation may be delayed with cryptococcal disease, which is most commonly seen when the CD4 count is ≤100 cells/μL. A point-of-care assay for cryptococcal infection is currently being evaluated and will be included in the screening protocol once it is available to facilitate referral of participants for appropriate evaluation and management. The point-of-care cryptococcal antigen test uses a finger-stick sample of whole blood. Persons who screen positive for cryptococcal antigen will receive their results as part of their referral to the clinic for further evaluation and management.

##### Screening viral load and ART testing

Two dried blood spot (DBS) cards will be collected at screening and stored for testing for HIV viral load and antiretroviral therapy. The maximum volume of blood to be collected is 0.5mL [2 spots on the first card and three spots on the second]. The cards will be stored at the study sites under appropriate conditions. If validated viral load testing through point-of-care viral load testing is available, it may be used instead of DBS.

##### Volume of specimens

For most participants for whom point-of-care tests will be used, 1.5 mL of blood will be collected. A maximum of 12mL of blood will be collected for testing for CD4, creatinine, DBS, and viral load including specimens for QA/QC. The specimens will be disposed of after testing.

##### Chart abstraction

For all study groups, engagement in care (clinic visits, ART initiation, and pharmacy refills) may be abstracted from clinic records or ART refills confirmed by participant report and/or staff member confirming ART supplies either in person or via phone/telehealth.

#### Six-month visit

##### Specimen collection

Participants will have specimens collected for ART monitoring at the month 6 visit, specifically creatinine and viral load. Volumes and frequency of laboratory monitoring are described below.

##### Community (smart locker) delivery

If participants are randomized to the community (smart locker) delivery group, they will receive ART refill delivery via a smart locker in a secure location. They will complete their clinical screen via telehealth, including an option for a secure video link, and self-collect specimens for viral load screening. If the participant is not able to access the smart locker due to the geographic location, mobile smart lockers will be provided via a mobile van at the time of delivery.

##### Home ART delivery

Participants randomized to the home ART delivery group will receive ART delivery at home or at a location of their choice, such as a community center. At ART delivery visits, a clinical screening questionnaire will be completed, and blood collected for viral load and creatinine tested as indicated by the clinical monitoring schedule following local guidelines. These procedures will be conducted by a study team member in person, with oversight from a nurse, or via telehealth with a nurse. If no-contact delivery is necessary due to COVID-19 restrictions, the participant will self-collect DBS for viral load testing and provide that when the medication is delivered. *Delivery questionnaire*: A core set of delivery questions will be administered, including the location of home and work, delivery preferences, availability to receive delivery, and preferred delivery window. Participants will have a home delivery appointment scheduled 3 months after ART initiation.

#### Follow-up (9, 12, and 15 months)

##### Follow-up procedures for participants in the home ART group

Follow-up visits at months 9, 12, and 15 will take place at the home or a location of the participant’s choice. Appointments for the home delivery, i.e., date and time that it will be available at home or a location easily accessible to the participants for follow-up, will be made at the time of ART initiation. Appointments will be made prior to ART supply running out, and confidential, neutral text message reminders will be provided for participants who have access to private messaging and phone calls. Participants will be able to reschedule their appointments by text message. Contact information will be provided for study staff, who participants can contact with questions. Clinic details will be provided to the participant who can contact the clinic at any time.

At the follow-up visits, participants will have their identification confirmed and GPS location collected, complete a questionnaire including adverse event and social harm screening, complete labs for ART and TPT monitoring, and collect ART resupply, and counselors will answer questions and provide support for HIV care. Participants will have their blood pressure measured if indicated. Participants will receive counseling and be referred to local clinics for further evaluation and treatment as needed. Participants will complete a questionnaire on clinical review, their experience accessing care, including barriers to care, linkage to care, uptake of ART, adherence, adverse events, lab testing, social harms, risk behavior, cost of engaging in care, HIV clinical care, and knowledge about HIV treatment for clinical and prevention benefits. Deliveries may be conducted as no-contact deliveries, with visits via telehealth and self-collected DBS specimens for monitoring if COVID-19 restrictions or other limitations on mobility are put in place.

##### Follow-up procedures for participants in the community (smart locker) group

Follow-up visits at months 9, 12, and 15 will take place via telehealth with ART provided at the smart locker and the participant depositing their self-collected specimens at the smart locker for collection. Smart locker ART pick-up windows will be made for ART refills and monitoring prior to their ART supply running out, and confidential, neutral text message reminders will be provided for participants who have access to private messaging and phone calls. Participants will be able to reschedule their telehealth appointments and locker pick-up by text message. Contact information will be provided for study staff, who participants can contact with questions. Clinic details will be provided to the participant who can contact the clinic at any time. With each ART delivery, the following procedures will happen:

- Collection of the GPS coordinate of the delivery location
- ART, tuberculosis preventive treatment (TPT), and other chronic meds will be dispensed
- Schedule next delivery or visit as indicated
- Participants will have refills every 3 months
- Health questionnaires will be completed
- Blood draw or self-collected DBS (if necessary, for POC creatinine and viral load (up to 12.5mL))
- Participants may have their medical records reviewed by the study team to record any visits to the clinic and the reason

##### Follow-up procedures for ART monitoring in the community (smart locker) and home delivery groups

Blood specimens will be collected for viral load and creatinine monitoring according to national guidelines. In the smart locker group, participants will self-collect DBS for viral load testing. In the home ART delivery group, specimens will either be collected by staff or, for no contact deliveries, by participants. For participants on fixed dose combination TDF/3TC/DGT, the first-line regimen in South Africa, safety labs will consist of viral load monitoring at months 6 and 12 and creatinine monitoring following local guidelines. Creatinine monitoring will be conducted using the StatSensor. If the StatSensor is not available, 5mL of blood will be collected for creatinine testing in the lab and the result made available to the participant. Participants with significantly raised creatinine levels (an increase of >0.5 mg/dL) will be referred to the clinic for investigation and management.

HIV viral load testing will use the gold standard PCR viral load test, using plasma or DBS specimen) or the validated Xpert assay. The viral load result will be made available to the participant, with counseling to help with the interpretation of the result.

Participants will receive a non-identifying text message within 1 week to communicate that “all is going well” (i.e., if their viral load is suppressed), contacting them to have additional adherence counseling—“contact us for more information” (i.e., if first viral load not suppressed), or asking them to come into the clinic for assessment for treatment failure (i.e., if two viral loads are not suppressed following intervening adherence counseling). Adherence counseling for participants with VL > 1500 copies/mL will identify barriers and strategies to increase ART adherence.

If indicated by local guidelines, cotrimoxazole (Bactrim) and/or isoniazid prophylaxis will be provided to participants following national guidelines and for whom it is not contra-indicated. At each study visit, female participants will be asked about pregnancy and referred for antenatal care if they report missed periods and/or signs or symptoms of pregnancy. They will be encouraged to remain on ART to decrease the risk for mother to child transmission of HIV. Pregnant participants are eligible to remain in the study and will continue to have follow-up visits.

##### Follow-up procedures for the SOC clinic group (with and without lottery incentives)

Participants will initiate ART at the clinic and, if feasible, receive quarterly chart review (either paper charts or through electronic medical records (EMRs) where available) to review safety labs (creatinine at 3, 6, and 12 months after ART initiation, for participants on TDF, and viral load 6 and 12 months after ART initiation), adherence data, ART refills, and engagement in clinical care. A study team member will facilitate linkages of the participant to the study to enable follow-up and chart extraction. Participants will complete a questionnaire on opportunistic infections, engagement in care, adverse events, and, if they have started ART, ART symptoms either by phone or in person at the clinic at months 3, 6, 9, 12, and 15. Participants will receive an 18-month follow-up visit during which they will complete the follow-up questionnaire (month 18 visit detailed below).

#### Procedures for 18-month or exit visit for all participants

Follow-up visits at month 18 or exit will take place in person at the mobile van, home, or clinic, by participant choice. At the follow-up visits, counselors will answer questions and provide support for HIV care. Participants will complete a questionnaire on their experience accessing care, including barriers to care, linkage to care, uptake of ART, adherence, adverse events, reasons for loss to follow-up, CD4 and viral load testing, social harms, risk behavior, cost of engaging in care, HIV clinical care including co-infections, and knowledge of HIV treatment and prevention. Examination of HIV care documentation (e.g., registration card from HIV care clinic) and recording of medications (e.g., HIV care medications and ART) will take place. If feasible, chart extraction will be conducted to record engagement in HIV care and lab monitoring for all participants. A maximum of 11 mL of blood (2 teaspoons) will be collected for viral load and creatinine testing. The specimens will be disposed of after testing has taken place. HIV viral load testing will use the gold standard PCR viral load test or the validated alternate approach. The viral load result will be made available to the participant, with counseling to help with the interpretation of the result.

### Plans to promote participant retention and complete follow-up {18b}

Screening and enrolment will be conducted in person and will be followed by quarterly visits thereafter for 18 months. Participants will also receive a phone call 7 days and 30 days after initiating ART to complete a standard symptom screen for ART adverse effects and referred to clinic care if necessary. If indicated, tuberculosis (TB) preventive treatment (TPT) and trimethoprim-sulfamethoxazole will be provided to prevent TB and pneumocystis pneumonia. Tracking and tracing activities will be undertaken by the community engagement team who will keep an active presence in study communities for the duration of the trial. Every effort will be made to retain all participants at the 18-month exit visit to ensure high-quality data and that continuity of care can be put in place before the study exit. We will follow well-established practices of notifying participants of their study follow-up visits via SMS and WhatsApp messaging and telephone calls.

### Data management {19}

Screening and enrolment questionnaires will be administered to all participants using REDCap (Research Electronic Data Capture). REDCap was developed through a National Institutes of Health grant to Vanderbilt University. It is free and consists of a secure web-based application and associated mobile phone app. It is used globally to support data capture for research studies and provides a graphical interface for survey development, data entry validation, audit trails and a change log of all data edits, and the ability to export data in formats that can be easily read by common statistical packages. REDCap uses role permissions to ensure only authorized staff have access to appropriate data and functions.

The HIV questionnaires are also available on REDCap and consist of questions which will be administered to persons living with HIV identified through HIV testing. Checks will be placed to ensure correct information has been entered. Data from laboratory testing (e.g., plasma HIV viral load) will be collected by participant ID number and merged into the database.

All questionnaires will be loaded onto the REDCap mobile app which will allow for offline data collection. Both the mobile phone and REDCap are password protected to ensure data safety in the event a handset is lost. Surveys are then uploaded to the secured UW server. The de facto standard for securing network traffic is Secure Sockets Layering (SSL). This technology is fully supported by the handsets used in this study and ensures that all data transferred between the device and the server is encrypted. Similarly, when reviewing, exporting or managing data all communications between browser and server are encrypted. All data will be encrypted. Servers are secured by firewalls to prevent unauthorized access and denial of service attacks. Data is protected from virus threats using anti-virus technology. The study database will be backed-up regularly.

### Confidentiality {27}

All participants will go through an informed consent process. The study staff will provide information for those who are interested in being tested but not interested in study participation. All potential clients will read or have read to them the consent form. The consent form will be available in both the local language and English, and participants will select which language they prefer to be consented in. Each participant will be given an opportunity to ask questions about study participation. If a participant agrees to participate in the study, the study staff will obtain electronic consent, with paper consents as a backup in case of electronic devices not being available. After consenting to participate, a participant may voluntarily withdraw from the study at any time and can choose not to have his/her responses submitted to the study team. Staff have undergone training on confidentiality, couple counseling, and testing and have substantial experience conducting VCT and home HTC. All staff members sign confidentiality agreements. For pre-screening, risks are associated with loss of confidentiality and finger-stick blood collection, including pain, fainting, and rarely, infection or bruising. The risk of loss of confidentiality is minimized due to participant control over the decision of where and when to test using self-administered kits. HIV self-administered testing has been shown to be highly acceptable in other settings in sub-Saharan Africa. However, as with all HIV testing, psychological distress is a risk for individuals who test positive for HIV. Since self-administered testing may be done alone by the participant, lack of immediate support and interpretation of results may contribute to distress. Pre-screening self-testing kits will include contact details to study staff for post-screening counseling (to return for either in-person or text message/telephonic support). Study staff will be available to provide counseling and referral to minimize psychological distress following self-administered pre-screening.

### Plans for collection, laboratory evaluation, and storage of biological specimens for genetic or molecular analysis in this trial/future use {33}

To ensure the confidentiality of participants, all data will be coded by subject number. Data recorded on paper will be kept in locked cabinets with access only by members of the research team. There will be strict limited access to electronically stored data as well, using password protection. Research records will be kept confidential following national regulations. The subject’s name or other personal identifiers will not be included with specimens sent to the laboratory, which will be identified only by a code number. Interviewers and support staff will be trained on procedures for maintaining confidentiality. Text messages will be neutral and not contain information about the participant’s HIV status, e.g., appointment reminders will say “Your appointment is on Tuesday at 3pm”. At each visit, staff will ask about social harms, complete a detailed report, link the participant to community resources for support, and report social harms to the ethics review board. If multiple social harms are observed, the study procedures and text message content will be reviewed by the study team and community advisory boards and recommended changes submitted to regulatory bodies for review.

The recorded in-depth interviews will be transcribed and, for interviews conducted in isiZulu, then translated to English. As part of the transcription and translation, investigators will delete any identifying information to ensure the final transcripts protect confidentiality. Recordings will be stored in password-protected files on a secure, firewalled server at the HSRC and the study site in South Africa. Only the research team, the translator, and the transcriptionist will be permitted access.

## Statistical methods

### Statistical methods for primary and secondary outcomes {20a}

Statistical analysis is summarized below. Additional details will be provided in a statistical analysis plan (SAP) prior to the analysis of the trial data.

#### Primary outcome

We will assess the contribution of lottery incentives to the primary outcome of viral suppression after 18 months by comparing outcomes for individuals in two of the embedded adaptive interventions—(i) start with best clinic practices and continue for 18 months and (ii) start with best clinic practices plus lottery incentives and continue for 18 months—in a logistic regression model adjusted for age and sex. The study subjects who inform this comparison are those who are virally suppressed at 6 months and those who are not virally suppressed at 6 months but are randomized to continue in their baseline treatment. Following Nahum-Shani et al. [24], we will account for the second-stage randomization by duplicating data for the second group of subjects and using generalized estimating equations (GEE) with an independence working model to account for the resultant correlation [25].

We will assess the relative contributions of home- and community-based ART treatment to the primary outcome of viral suppression by comparing outcomes for individuals not virally suppressed after 6 months who are randomized to these two interventions in the second stage. We will estimate the odds ratio comparing the effectiveness of these two interventions relative to clinic-based care using multivariate logistic regression adjusted for age and sex. Subjects with baseline clinic-based interventions that do and do not include lottery incentives will be combined. For all analyses, we will estimate odds ratios, 95% confidence intervals, and *p*-values with robust standard errors [25]. We will first test for superiority for the primary outcome of viral suppression. If the intervention is superior, the analysis will stop. If the intervention is not superior, we will test for non-inferiority with a conservative non-inferiority margin of 5%.

#### Secondary outcomes

The same analyses as outlined for the primary outcome of viral suppression will be repeated for the secondary outcomes of retention in care (defined as the proportion of clinical visits and medication refills missed over the last 12 months of the intervention) and time to ART initiation, where logistic regression will be replaced by linear regression and Cox proportional hazards analysis, respectively.

#### Testing for non-inferiority in addition to superiority

We hypothesize that community-based/home ART initiation will be acceptable and have a higher or comparable impact on the clinical outcome of HIV viral suppression compared to the standard clinic ART delivery model. For primary and secondary outcomes, we will first test for superiority. If the intervention is superior, the analysis will stop. If the intervention is not superior, we will test for non-inferiority with a conservative non-inferiority margin of 5%.

### Interim analyses {21b}

Interim analyses will include summaries of demographic data and whether participants were living with HIV who have a detectable viral load at enrollment or were not engaged in care at baseline. We will also summarize the numbers of adverse events and individuals lost to follow-up. All summaries will be conducted overall and broken down by randomization group (first- and second-stage randomization). These summaries will be calculated at times that align with meetings of the DSMB and will be shared with the DSMB. Aside from group-specific information about adverse events and loss to follow-up, summaries will also be shared with the SMART ART investigator team. Operational futility will be monitored to ensure trial resources are not squandered.

### Methods for additional analyses (e.g., subgroup analyses) {20b}

We will conduct subgroup analyses for the primary and secondary outcomes where we separate individuals living with HIV who have a detectable viral load at enrollment and individuals not engaged in care at enrollment. We will conduct further subgroup analyses for all primary and secondary outcomes restricted to male/female participants and restricted to participants older/younger than 40 years. We will use product term interaction models to determine if there are statistically different associations between the outcomes based on sex or age.

An exploratory analysis will estimate the clinic-specific rates of viral suppression and retention in care using multivariate logistic and linear regression models, respectively, with dummy variables for clinic and adjusted for age and sex in order to assess clinic heterogeneity. We will derive clinic-specific point estimates and 95% confidence intervals and will also calculate overall *p*-values for differences between clinics. As a sensitivity analysis, we will repeat all analyses for primary and secondary outcomes using GEE with an independence working model to account for correlation between participants recruited from the same clinic.

### Methods in analysis to handle protocol non-adherence and any statistical methods to handle missing data {20c}

The primary analysis will be modified intent-to-treat (mITT) restricted to participants who are not lost to follow-up and have complete endpoint data at the 18-month follow-up (inclusion in the mITT analysis will be determined separately for each outcome). As a sensitivity study, we will repeat all analyses as intent-to-treat (ITT), including all randomized participants and imputing missing endpoint data as follows: (i) individuals lost to follow-up will be assumed not to be virally suppressed, (ii) individuals lost to follow-up prior to initiating ART will be assumed to have never initiated ART, and (iii) for individuals lost to follow-up, the proportion of missed clinic visits and medication follow-ups will be calculated for all available months in the final 12 months of follow-up and will be assumed to be one in the remaining months.

### Plans to give access to the full protocol, participant-level data, and statistical code {31c}

Using a data sharing platform, a complete dataset with patient identifies removed that is sufficient to reproduce the study findings will be made available approximately 1 year after completion of the trial. To gain access, a concept sheet summarizing the analyses to be done will first need to be submitted to the principal investigators for review. Further inquiries can be directed to the SMART ART Scientific Committee at avanheerden@hsrc.ac.za.

## Oversight and monitoring

### Composition of the coordinating center and trial steering committee {5d}

The SMART ART study will be jointly led by Harvard University and the Center for Community Based Research, Human Sciences Research Council of South Africa.

### Composition of the data monitoring committee, its role, and reporting structure {21a}

We will constitute a Data Safety and Monitoring Board (DSMB) who will meet every 6 months to review the available study data. The analysis will evaluate participant safety (SAEs and social harms) and available endpoint data and review the operational factors, specifically participant enrollment and follow-up, to assess safety and study execution. To examine whether the study design should be modified, we will conduct a conditional power analysis of the process outcomes (ART uptake and adherence) and early endpoint data (viral suppression) to assess the futility for each of the two treatment groups. We will compare the frequency of ART-related SAEs between the three study groups to assess safety, and review the serious adverse events to see if any could be prevented.

### Adverse event reporting and harms {22}

All participants who experience adverse events will receive follow-up until the adverse event is resolved. The SMART ART study clinical monitor, based at the University of Washington Coordinating Center, will review all severe (grade 3/4) and serious adverse events to ensure follow-up and reporting. Serious adverse events (SAEs) will be documented and reported to involved regulatory bodies per their regulations. Serious adverse events will also be reported to the UW ICRC Coordinating Centre Clinical Monitor, who will track the events until resolved. SAE reports will be reviewed to determine if the SAE could have been avoided.

Participants who initiate ART and who will collect ART refills from the mobile van will receive a phone call 1 week and 30 days after ART initiation to enquire about adverse events and refer participants to the clinic for evaluation if necessary. During participant ART resupply and monitoring visits, they will complete a standardized symptom screening questionnaire for adverse effects of ART, tuberculosis, opportunistic infections, and STIs. Furthermore, all participants on TDF/FTC or 3TC/EFV will receive the standard ART screening, including a creatinine test to monitor their renal function, at mobile van visits. Clinical side effects of TDF/FTC or 3TC/EFV include gastrointestinal disturbance (nausea and vomiting) and vivid dreams. Participants will be referred to the clinic for symptoms or signs requiring further investigation and/or treatment. We have established relationships with the clinics who expect to see study participants.

All cases of social harm for participants will be documented. To ensure we are aware of all cases of adverse events associated with HIV results, we will ask each participant specifically about any social harm experienced as a result of learning HIV results at the follow-up visits. Staff will gather additional information about social harm for reports. Study staff are well versed in community-based organizations where individuals and couples can access on-going psychological support and help in case of gender violence and will provide information about these organizations to individuals and couples as needed. In addition to providing specific counseling, staff will refer participants for support to local organizations. The study teams have trained staff able to handle crisis situations due to couple conflict or difficulties in coping with positive HIV test results.

### Frequency and plans for auditing trial conduct {23}

The project management group will meet weekly to review key metrics including balanced randomization, screen to enrollment rate, weekly recruitment, and retention rates. This team will be supported by a Data Safety and Monitoring Board (DSMB) and Institutional Review Board (IRB). The DSMB will meet annually for about 2 h per meeting with additional time for review of the DSMB report prior to the meeting. The IRB require that the study comply with ICH-GCP guidelines and provide annual review and renewal.

### Plans for communicating important protocol amendments to relevant parties (e.g., trial participants, ethical committees) {25}

Modifications to study protocol, informed consent forms, and recruitment materials will all be submitted for review to both the HSRC and Harvard IRBs. The study will be reviewed annually by both IRBs when relevant information about recruitment, enrolment, follow-up, AEs and SAEs, and protocol deviations and violations are submitted as a unified report.

## Dissemination plans {31a}

Our team of investigators is fully committed to rapid and multi-pronged dissemination of study results, regardless of the results. Through our previous HIV prevention trials (HPTN 039, Partners in Prevention HSV/HIV Transmission Study, and Partners PrEP Study), we have found that community and stakeholder consultations are important at every stage of the research, spanning study concept to results, in order to build and sustain trust in research and research partnerships. Once results are available, we will provide timely results to the Umgungundlovu District and KZN Province in South Africa community stakeholders, South African Department of Health officials, and South African PEPFAR leadership. Once the study analysis is complete, data will be presented at local and international conferences and will be submitted for publication.

## Discussion

Globally, more than half of the world’s 37 million people living with HIV are on antiretroviral therapy (ART) representing immense and encouraging success with access to HIV care. ART prevents disease, death, and HIV transmission and HIV-positive persons can expect to live as long as their HIV-negative peers when their viral load is undetectable. However, treatment success still lags behind goals. In South Africa alone, 8 million HIV-positive persons require ART for life and only 4.5 million are currently on ART. Patient barriers to care, such as missed wages, transport costs, and long wait times for clinic visits and ART refills, are associated with detectable viral load, the hallmark of struggling to access and take ART. HIV differentiated service delivery (DSD) has simplified ART delivery: incentives, multi-month scripts, fast-track ART, and community or home ART delivery motivate clients, reduce the frequency of clinic visits, and decongest clinics. DSD is standard for clients who achieve viral suppression and engage in care; however, DSD needs adaptation to serve clients who are not succeeding. Indeed, persons who are not engaged in care arguably need simplified, client-centered approaches even more than those who can successfully engage.

This study will build on the investment, experience, results, and strong partners established through the Lotto-to-Link, DO ART, and Deliver Health Studies and test the impact of adaptive strategies for persons not suppressed on ART/not engaged in care, in sequence, to best clinic practices with or without lottery incentives, community-based ART, and home delivery. A suite of adaptive DSD strategies, including community-based ART, have been tested among stable clients with viral suppression. Lottery incentives effectively change short-term behavior, increasing ART initiation. Community and home ART delivery increases ART coverage and simplifies ART access overcoming clinic barriers. For stable clients, these DSD activities are as effective as clinic-based care in terms of achieving and maintaining viral suppression, although among stable clients they have not shown superiority in viral suppression or cost savings. In contrast, DSD has the potential to improve rates of viral suppression and retention in care and save costs among more hard-to-reach groups. There is great potential that DSD systems can be client-responsive and system-efficient for subgroups requiring additional services, matching services with client needs.

A sequential, comprehensive package of DSD approaches, with each step increasing the intensity of service provision—adaptive DSD—has not been tested to determine the proportion and characteristics of persons who would achieve viral suppression and retention in care and to estimate the cost-effectiveness and budget impact. A Sequential Multiple Assignment Randomized Trial (SMART) design facilitates the evaluation of a stepped, adaptive approach to achieving viral suppression with “right-sized” interventions Adapting ART delivery and support interventions will require buy-in from clients and providers to understand what works and why. Clinic services are changing to become more welcoming to clients re-engaging in care. Through this study, we will generate evidence on which strategies increase viral suppression.

## Trial status

This protocol references version 1.2 from July 9, 2021. The first participant was screened on November 3, 2021. The first participant was enrolled on November 10, 2021. The study is expected to conclude recruitment on April 24, 2023, with all enrolled participants followed quarterly for 18 months thereafter.

## Data Availability

Listed investigators have full access to the final dataset. The final dataset will be curated and made available on request.

## Abbreviations

3TC: Lamivudine
AE: Adverse event
ART: Antiretroviral therapy
CO^2^: Carbon dioxide
COVID-19: Coronavirus disease 2019
DAIDS: Division of AIDS
DO ART: Delivery optimization of antiretroviral therapy
DSD: Differentiated service delivery
DSMB: Data Safety and Monitoring Board
EFV: Efavirenz
FTC: Emtricitabine
GEE: Generalized estimating equations
HIVST: HIV self-testing
HPTN: HIV Prevention Trials Network
HSRC: Human Sciences Research Council
HTC: HIV testing and counseling
ICH: International Council for Harmonisation
ICRC: International Clinical Research Center
IRB: Institutional Review Board
KZN: KwaZulu-Natal
LTFU: Lost to follow-up
MSF: Médecins Sans Frontières
NDOH: National Department of Health
PEPFAR: United States President’s Emergency Plan for AIDS Relief
PrEP: Preexposure prophylaxis
REDCap: Research Electronic Data Capture
SAE: Serious adverse event
SMART: Sequential Multiple Assignment Randomized Trial
SOC: Standard of care
STI: Sexually transmitted infection
TB: Tuberculosis
TDF: Tenofovir disoproxil fumarate
TPT: TB preventive treatment
UW: University of Washington, Seattle
VL: Viral load
